# A retrospective study of strabismus surgery in a tertiary eye hospital in the Chaoshan area in China from 2014 to 2020

**DOI:** 10.1186/s12886-022-02479-8

**Published:** 2022-06-04

**Authors:** Yu Bi, Jason C. Yam, Shibin Lin

**Affiliations:** 1 Joint Shantou International Eye Center of Shantou University and the Chinese University of Hong Kong, Guangdong Province 515041 Shantou, China; 2 Department of Ophthalmology and Visual Sciences, The Chinese University of Hong Kong, Hong Kong, Hong Kong

**Keywords:** Distribution, Retrospective survey, Strabismus, Surgery

## Abstract

**Background:**

To investigate the distribution pattern of strabismus surgery at an eye center in southern China.

**Methods:**

The data of all patients who underwent strabismus surgery at the Joint Shantou International Eye Center of Shantou University /Chinese University of Hong Kong from 2014 to 2020 were retrospectively analyzed. The type of strabismus and its relationship with age and sex were analyzed, and the trend in the number of strabismus surgeries was observed.

**Results:**

The data of 4640 patients included a total of 5,282 surgeries.

Exotropia surgery was the most common, accounting for 54.0% of all strabismus surgeries, which tended to increase over time, but not significantly (*P* = 0.109). Constant exotropia was the most common exotropia, but its proportion decreased year by year. The next most common was intermittent exotropia. The percentage of surgeries for intermittent exotropia increased from 2016 to 2020. Surgery for esotropia accounted for 22.1% of all strabismus surgeries and became significantly less common from 2016 to 2020 (*P* < 0.01). Among patients with intermittent or constant exotropia, the proportion of patients younger than 12 years who underwent surgical intervention increased yearly, while this proportion amoung patients older 12 years old with constant exotropia decreased yearly.

**Conclusion:**

Among exotropia surgeries, surgery for constant exotropia was the most common but decreased in prevalence annually. Children under 12 years old were the most affected population.

## Background

Ocular misalignment may inhibit the development of binocular vision. It may lead to amblyopia, which causes permanent vision deficits if not treated early. Timely intervention may be required to ensure optimal visual function [1, 2]. Strabismus also has a negative psychosocial impact on children, and the parents of these children may also experience a decline in quality of life [3].

There are many reports on the prevalence and distribution of strabismus, but epidemiological studies of strabismus have been primarily conducted in Western populations. There have also been some studies in Asian countries, but they have reached different conclusions than those done in Western countries. Esotropia is the most common in Western countries, and the number of children receiving surgical correction of their strabismus appears to be declining [4, 5]. Exotropia is the most common in Asian countries, and the number of surgeries for it is increasing [6–8]. The aim of this study is to determine which kind of strabismus is most common in the Chinese population treated at our eye center and to calculate the age distribution of these patients and the proportion with each type.

## Methods

The data of 4640 patients who underwent strabismus surgery in the Department of Strabismus at the Joint Shantou International Eye Center of Shantou University/Chinese University of Hong Kong (JSIEC) from January 2014 to December 2020 were retrospectively analyzed. All clinical records were provided by the central information system of the JSIEC. The information included the name, sex, age, and diagnosis of each patient. The diagnosis and classification of strabismus were based on an expert consensus on the classification of strabismus (Strabismus and Pediatric Ophthalmology Group of the Ophthalmology Branch of the Chinese Medical Association, 2015) [9]. The uncorrected visual acuity and best-corrected visual acuity of the patients were assessed and recorded during hospitalization. They all underwent autorefraction, slit-lamp microscopy, direct ophthalmoscopy, and noncontact tonometry. The results of the Hirschberg test, the alternate cover test, eye movement analysis, and synoptophore were recorded. Stereopsis was examined using a TNO chart. Strabismus measurements were performed using the alternate prism cover test, at 33 cm for near fixation and at 6 m for distance fixation. For children with perceptual strabismus and low visual acuity in one eye and for some children who were unable to cooperate, the Krimsky method was used for measurement.

Statistical analysis was performed using the software package SPSS 20.0. Fisher’s exact test and the chi-squared test were used to compare the proportions between groups. The results were considered statistically significant when *p* < 0.05.

## Results

The data of the patients who underwent strabismus surgery at the JSIEC from January 2014 to December 2020 were collated and analyzed. A total of 4640 patients, aged 1 to 73 years, with a mean age of 15.9 ± 9.5 years, were included. There were 2330 males (50.2%) and 2310 females (49.8%); there was no significant male or female skew (χ^2^ = 3.344, *P* = 0.765) (Table 1).

**Table 1:** Gender distribution of strabismus surgery

Year	Male	Female
2014	204 (50.8%)	197 (49.1%)
2015	292 (50.6%)	285 (49.4%)
2016	290 (51.0%)	279 (49.0%)
2017	344 (52.4%)	312 (47.6%)
2018	401 (49.0%)	417 (51.0%)
2019	447 (48.3%)	478 (51.7%)
2020	352 (50.7%)	342 (49.3%)
Total	2330(50.2%)	2310(49.8%)
χ2	3.344	
*P*	0.765	

There were a total of 5282 surgeries. Most of the surgeries were exotropia surgery [2854 (54.0%) cases], followed by esotropia surgery [1136 (21.5%) cases]. The proportion of esotropia surgeries showed a decreasing trend from 2016 to 2020 (χ^2^ = 31.95, *P* < 0.01), while the proportion of exotropia surgeries showed an increasing trend over time, but this was not significant (*P* = 0.109) (Table 2). Among surgeries for exotropia, surgeries for constant exotropia were the most common, accounting for approximately 54.3% of them, though they decreased from 75.3% in 2014 to 31.7% in 2020 (χ^2^ = 213.44, *P* < 0.01). Second most common were surgeries for intermittent exotropia, accounting for approximately 36.5% of all surgeries for exotropia, though their increased from 19.9% in 2014 to 63.3% in 2020 (χ^2^ = 266.273, *P* < 0.01) (Table 3).

**Table 2:** Distribution of strabismus types in surgical patients from 2014 to 2020

Year	Esotropia	Exotropia	Vertical and torsional strabismus	Special types of strabismus
2014	140 (27.9%)	251 (50.0%)	102 (20.3%)	9 (1.8%)
2015	139 (21.4%)	364 (56.0%)	125 (19.2%)	22 (3.4%)
2016	151 (24.2%)	322 (51.7%)	127 (20.4%)	23 (3.7%)
2017	163 (23.7%)	360 (52.3%)	118 (17.2%)	47 (6.8%)
2018	211 (22.0%)	516 (53.8%)	152 (15.8%)	80 (8.3%)
2019	199 (18.3%)	599 (55.0%)	205 (18.8%)	87 (8.0%)
2020	133 (17.3%)	442 (57.4%)	99 (12.9%)	96(12.5%)
χ2	31.950	10.402	22.270	85.217
*P*	< 0.001	0.109	0.01	< 0.001

**Table 3:** Distribution of exotropia in surgical patients from 2014 to 2020

Year	Intermittent exotropia	Constant exotropia	Sensory exotropia	After strabismus surgery	Non concomitant exotropia
2014	50 (19.9%)	189 (75.3%)	3 (1.2%)	6 (2.3%)	3 (1.2%)
2015	88 (24.2%)	248 (68.1%)	18 (4.9%)	3 (0.8%)	7 (1.9%)
2016	70 (21.7%)	191 (59.3%)	47(14.6%)	9 (2.8%)	5 (1.6%)
2017	127 (35.3%)	198 (55.0%)	24 (6.7%)	10 (2.8%)	1 (0.3%)
2018	200 (38.8%)	265 (51.4%)	24 (4.7%)	20 (3.9%)	7 (1.4%)
2019	312 (52.1%)	234 (39.1%)	25 (4.2%)	20 (3.3%)	8 (1.3%)
2020	280 (63.3%)	140 (31.7%)	13 (2.9%)	5(1.1%)	4 (0.9%)
χ2	266.273	213.440	70.052	13.368	
*P*	< 0.001	< 0.001	< 0.001	0.038	0.484^b^

^b^Fisher’s exact test.After strabismus surgery includes consecutive exotropia and residual exotropia

Among surgeries for esotropia, surgeries for non-accommodative esotropia were the most common, accounting for 63.8% of them, which did not change significantly over time (*P* = 0.84). Surgeries for partially accommodative esotropia accounted for 19.5% of all surgeries for esotropia, and their proportion increased from 15% in 2014 to 23.3% in 2020; however, the difference was not significant (*P* = 0.558) (Table 4). Among all the vertical and torsional strabismus surgeries, the most common type was inferior oblique overaction which accounted for approximately 60.9% of all surgeries for vertical and torsional strabismus.There was a significant difference in the composition of vertical and torsional strabismus from 2014 to 2020 (χ^2^ = 22.2, *P* < 0.05) (Tables 2 and 5). The percentage of cases of some special forms of strabismus showed a small upward trend, from 1.8% in 2014 to 12.5% in 2020,and the difference was significant (χ^2^ = 85.2, *P* < 0.01) (Table 2). A-V syndrome and dissociated vertical divergence were the more common special types of strabismus (Table 6).

**Table 4:** Distribution of esotropia in surgical patients from 2014 to 2020

Year	Partially accommodative esotropia	Non-accommodative esotropia	Sensory esotropia	After strabismus surgery	Non concomitant esotropia
2014	21 (15.0%)	92 (65.7%)	12 (8.6%)	4 (2.9%)	11 (7.9%)
2015	23 (16.5%)	89 (64.0%)	12 (8.6%)	5 (3.6%)	10 (7.2%)
2016	28 (18.5%)	94 (62.3%)	21 (13.9%)	5 (3.3%)	3 (2.0%)
2017	33 (20.2%)	109 (66.9%)	8 (4.9%)	11 (6.7)	2 (1.2%)
2018	45 (21.3%)	132 (62.6%)	18 (8.5%)	9 (4.3%)	7 (3.3%)
2019	43 (21.6%)	119 (59.8%)	11 (5.5%)	15 (7.5%)	11 (5.5%)
2020	31 (23.3%)	87 (65.4%)	5 (3.8%)	6 (4.5%)	4 (3.0%)
χ2	4.889	2.681	14.797	7.041	14.833
*P*	0.558	0.848	0.022	0.317	0.022

After strabismus surgery includes consecutive exotropia and residual exotropia

**Table 5:** Distribution of vertical and torsional strabismus in surgical patients from 2014 to 2020

Year	Superior oblique overaction	Inferior oblique overaction	Superior oblique palsy	Inferior oblique palsy	Superior rectus palsy	Inferior rectus palsy
2014	39 (38.2%)	58 (56.9%)	3 (2.9%)		2 (2.0%)	
2015	44 (35.2%)	55 (44.0%)	24 (19.2%)	1 (0.8%)		1 (0.8%)
2016	20 (15.7%)	89 (70.1%)	17 (13.4%)		1 (0.8%)	
2017	19 (16.1%)	79 (66.9%)	19 (16.1%)			1 (0.8%)
2018	18 (11.8%)	108 (71.1%)	21 (13.8%)		2 (1.3%)	3 (2.0%)
2019	31 (15.1%)	133 (64.9%)	36 (17.6%)		3 (1.5%)	2 (0.9%)
2020	22 (22.2%)	52 (52.5%)	25 (25.3%)

**Table 6:** Distribution of other special types of strabismus in surgical patients from 2014 to 2020

Year	A and V syndrome	Dissociated vertical divergence	Duane’s retraction syndrome	Brown syndrome	Graves endocrine ophthalmopathy	Congenital fibrosis of the extraocular muscles	Strabismus fixus	Helveston syndrome	Nystagmus
2014		7 (77.8%)					1 (11.1%)		1 (11.1%)
2015		17 (77.4%)	1 (4.5%)		1 (4.5%)				3 (13.6%)
2016	1 (4.3%)	20 (87.0%)	1 (4.3%)				1 (4.3%)		
2017	18 (38.3%)	25 (53.2%)	1 (2.1%)			1 (2.1%)		1 (2.1%)	1 (2.1%)
2018	40 (50.0%)	37 (46.2%)	2 (2.5%)		1 (1.2%)				
2019	42 (48.3%)	33 (37.9%)	5 (5.7%)	1 (1.1%)	1 (1.1%)		2 (2.3%)		3 (3.4%)
2020	71 (74.0%)	15 (15.6%)	3 (3.1%)	2 (2.1%)	1 (1.0%)			1 (1.0%)	3 (3.1%)

The age distribution of strabismus surgeries changed significantly over the study period, children aged under 12 representing the largest group and showing an increasing trend over time (χ^2^ = 116.36, *P* < 0.01) (Table 7). The proportion of intermittent exotropia patients younger than 12 years old who underwent surgery has been significantly increasing in recent years(χ^2^ = 18.126, *P* < 0.01), accounting for 64.5% of all patients in 2020. The proportion of constant exotropia patients younger than 12 years old who underwent surgery also increased significantly (χ^2^ = 128.211, *P* < 0.01), accounting for 62.1% of all patients in 2020 (Tables 8 and 9).

**Table7:** Age distribution of strabismus surgery

Year	≤ 12	13–20	21–30	31–40	41–50	> 51
2014	113 (28.2%)	158 (39.4%)	104 (25.9%)	12 (2.9%)	6 (1.6%)	8 (2.0%)
2015	216 (37.4%)	160 (27.7%)	118 (20.5%)	33 (5.7%)	44 (7.6%)	6 (1.0%)
2016	230 (40.4%)	161 (28.3%)	132 (23.2%)	29 (5.1%)	11 (1.9%)	6 (1.1%)
2017	322 (49.1%)	178 (27.1%)	118 (18.0%)	22 (3.4%)	7 (1.1%)	9 (1.4%)
2018	403 (49.3%)	198 (24.2%)	159 (19.4%)	33 (4.0%)	17 (2.1%)	8 (1.0%)
2019	483 (52.2%)	236 (25.5%)	130 (14.1%)	44 (4.8%)	25 (2.7%)	7 (0.8%)
2020	379 (54.6%)	149 (21.5%)	127 (18.3%)	27 (3.9%)	8 (1.2%)	4 (0.6%)
χ2	116.36	47.109	35.049	7.668	74.755	
*P*	< 0.01	< 0.01	< 0.01	0.263	< 0.01	0.39^b^

**Table 8:** Age distribution of intermittent exotropia

Year	≤ 12	13–20	21–30	31–40	41–50	> 51
2014	21 (42.0%)	15 (30.0%)	12 (24.0%)	2 (4.0%)	0	0
2015	46 (52.3%)	28 (31.8%)	13 (14.8%)	1 (1.1%)	0	0
2016	33 (47.1%)	21 (30.0%)	14 (20.0%)	2 (2.9%)	0	0
2017	65 (51.2%)	35 (27.6%)	24 (18.9%)	3 (2.4%)	0	0
2018	111 (55.5%)	49 (24.5%)	29 (14.5%)	10 (5.0%)	1 (0.5%)	0
2019	182 (58.3%)	66 (21.2%)	32 (10.3%)	28 (9.0%)	4 (1.3%)	0
2020	183 (65.4%)	51 (18.2%)	35 (12.5%)	10 (3.6%)	1 (0.4%)	0
χ2	18.126	16.586	21.207	21.475	-	-
*P*	< 0.01	0.01	< 0.01	< 0.01	-	-

**Table 9:** Age distribution of constant exotropia

Year	≤ 12	13–20	21–30	31–40	41–50	> 51
2014	46 (24.3%)	75 (39.7%)	57 (30.2%)	7 (3.7%)	2 (1.1%)	2 (1.1%)
2015	90 (36.3%)	76 (30.6%)	63 (25.4%)	16 (6.5%)	2 (0.8%)	1 (0.4%)
2016	79 (41.4%)	65 (34.0%)	40 (20.9%)	5 (2.6%)	2 (1.0%)	0
2017	82 (41.4%)	53 (26.8%)	41 (20.7%)	15 (7.6%)	5 (2.5%)	2 (1.0%)
2018	134(50.6%)	72 (27.2%)	47 (17.7%)	8 (3.0%)	2 (0.8%)	2 (0.8%)
2019	158(67.5%)	45 (19.2%)	21 (8.9%)	6 (2.6%)	4 (1.7%)	0
2020	87(62.1%)	31 (22.1%)	15 (10.7%)	5 (3.6%)	2 (1.4%)	0
χ2	128.211	28.211	43.728	12.554	-	-
*P*	< 0.01	< 0.01	< 0.01	0.051	-	-

## Discussion

**Fig. 1:**
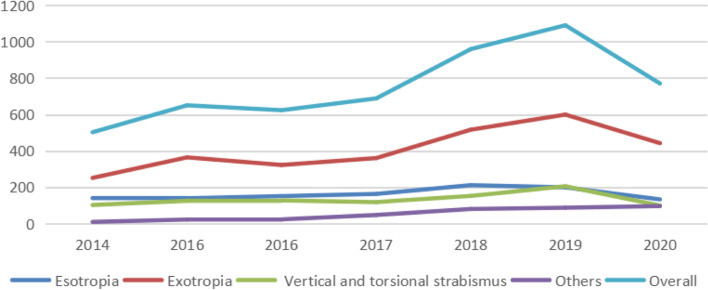
Distribution diagram of the number of surgical patients with each strabismus type from 2014 to 2020

In this study, we found that the number of strabismus surgeries in our hospital increased each year from 2014 to 2019 but decreased in 2020, a finding related to the COVID-19 pandemic in 2020 (Fig. 1). After the pandemic hit, the hospital took corresponding preventive measures, resulting in a decrease in the number of patients who underwent nonemergency surgery. We evaluated the various types of strabismus in our patients and found that the number of exotropia surgery was the most common, accounting for 54.0% of all strabismus surgeries. The following studies are consistent with our conclusions. Researchers at Beijing Tongren Hospital retrospectively analyzed all strabismus surgery data of the 4 years from 2003 to 2006 and found that the number of strabismus surgeries increased year by year, exotropia surgery being the most common. The same conclusion was reached in a similar study conducted by researchers at a hospital in Qingdao [7, 8].A study in Japan showed that the number of surgeries for exotropia was significantly greater than that for esotropia in both adult and pediatric patients [6]. In Singapore, the prevalence of strabismus in 6- to 72-month-old children was 0.80%, and the ratio of exotropia to esotropia was 7:1 [10]. Yu C et al. in Hong Kong observed 2704 patients with horizontal strabismus and found that exotropia was more common than esotropia [11].

However, Arora et al. reviewed strabismus surgery in children aged 0–16 years in England between 1994 and 2000 and found that the number of surgeries decreased by 41.2% [12]. MacEwen reviewed pediatric strabismus surgeries in the Tayside region of Scotland between 1986 and 2001 and found a 63% reduction in esotropia surgery in Scotland, including a 69% reduction in the Tayside region [13]. A study in the United Kingdom showed that the number of surgeries for children with strabismus aged 0 to 15 years fell significantly during a 10-year period but that the number of surgeries for esotropia was higher than that for exotropia [4]. Mba M et al. also found that esotropia surgery in the United States has decreased in recent years [14], while exotropia surgery has shown an upward trend year by year since 2016. Another study in the United States showed that between 1990 and 2009, although the number of surgeries decreased for esotropia and increased for exotropia, the overall number of surgeries for esotropia was still greater than that for exotropia [5]. Stidwill et al. in the United Kingdom observed 3075 patients with strabismus, and the ratio of esotropia to exotropia was 2.5–2.8: 1 [15]. An analysis of the various types of strabismus in pediatric patients in the Jeddah region of western Saudi Arabia showed that esotropia was predominant. Similar results were reported from Palestine by Medghalchi A and Mvogo et al. [16].

According to the above research, we may infer that the number of surgeries decreases in areas with a high rate of esotropia surgeries, and the same is true.

in areas with a high rate of exotropia surgeries. There are two reasons that could explain these result: (1) Most children with esotropia have refractive errors and amblyopia. Both early precision optics and improved eye adjustment function have a corrective effect on the eye position in esotropia, which can reduce the need for surgical intervention. (2) Surgery is the primary treatment for patients with exotropia [8].

The results of this study showed that constant exotropia was the most common exotropia but that its proportion decreased year by year. The second most common was intermittent exotropia. The percentage of surgeries for intermittent exotropia increased from 2016 to 2020. Some studies of Asian populations have shown that intermittent exotropia is the most common exotropia [7, 8]. This difference may be because the Chaoshan area is a third-tier city in China, its economic development and population education levels are relatively low compared with those of the first-tier cities, and residents’ knowledge about medical science is relatively lacking. Many patients with exotropia do not pay much attention to their intermittent exotropia, thus delaying treatment. In recent years, China has paid more attention to health education related to the prevention and control of myopia in children and adolescents and strengthened the screening of eye diseases in adolescents, providing an opportunity for intermittent exotropia to be detected and thereby improving the diagnosis of the disease.

None of the above studies mention the surgical age of patients with strabismus. The surgical age of patients can reflect the level of regional economy and population education, the extent of eye diseases examination in children, and the spread of popular science about related diseases. JSIEC mainly serves the Chaoshan area, including Shantou, Chaozhou, and Jieyang, among other places, with a permanent population of 14 million. This study found that 46.3% of the patients who underwent strabismus surgery were younger than 12 years, and this percentage showed an increasing trend over time. Because it is difficult for patients and their family members to remember the exact age of onset, the recorded age is the age at the time of surgery, not the age at which the disease started. In 2014, the age of patients undergoing strabismus surgery in this hospital was between 13 and 30 years old. Constant exotropia is the most common kind. For patients in this age group, surgery can correct the normal appearance only, but it is difficult to restore normal binocular function. The reason for such a poor prognosis is generally the parents’ lack of relevant knowledge, as well as deficient medical resources. Among the patients with either intermittent exotropia or constant exotropia who underwent surgery, the proportion of patients younger than 12 years old has been increasing, whereas the proportion of constant exotropia patients older than 12 years old who received surgical intervention has been decreasing. The exodeviations are related to the excessive divergence tone that may exceed the convergence tone, leading to drifting of eyes outwards [17]. The deviations often start as an exophoria. In this stage, the eyes are well aligned most of the time, and an exodeviation is noted only upon breaking the fusion with a cover test. In due course of time, these patients often progress to intermittent exotropia. The next stage is of constant exotropia, in which the patient is not able to fuse at a single object with both his eyes at one point [18]. Some intermittent exotropia patients will likely gradually develop constant exotropia, which may damage the use of visual function in young patients. Therefore, the decrease in constant exotropia patients older than 12 years old who underwent surgery may be related to the increase in intermittent exotropia patients younger than 12 years old who underwent surgical intervention.

In recent years, a strabismus/amblyopia prevention and treatment program has been gradually carried out throughout China, with an emphasis on childhood diseases and early intervention. This allows more patients to receive corresponding treatment in the early stage after the onset of strabismus, so the ages of strabismus patients who undergo surgical treatment has gradually fallen. The benefits of this program can also be seen in our study.

**Fig. 2:**
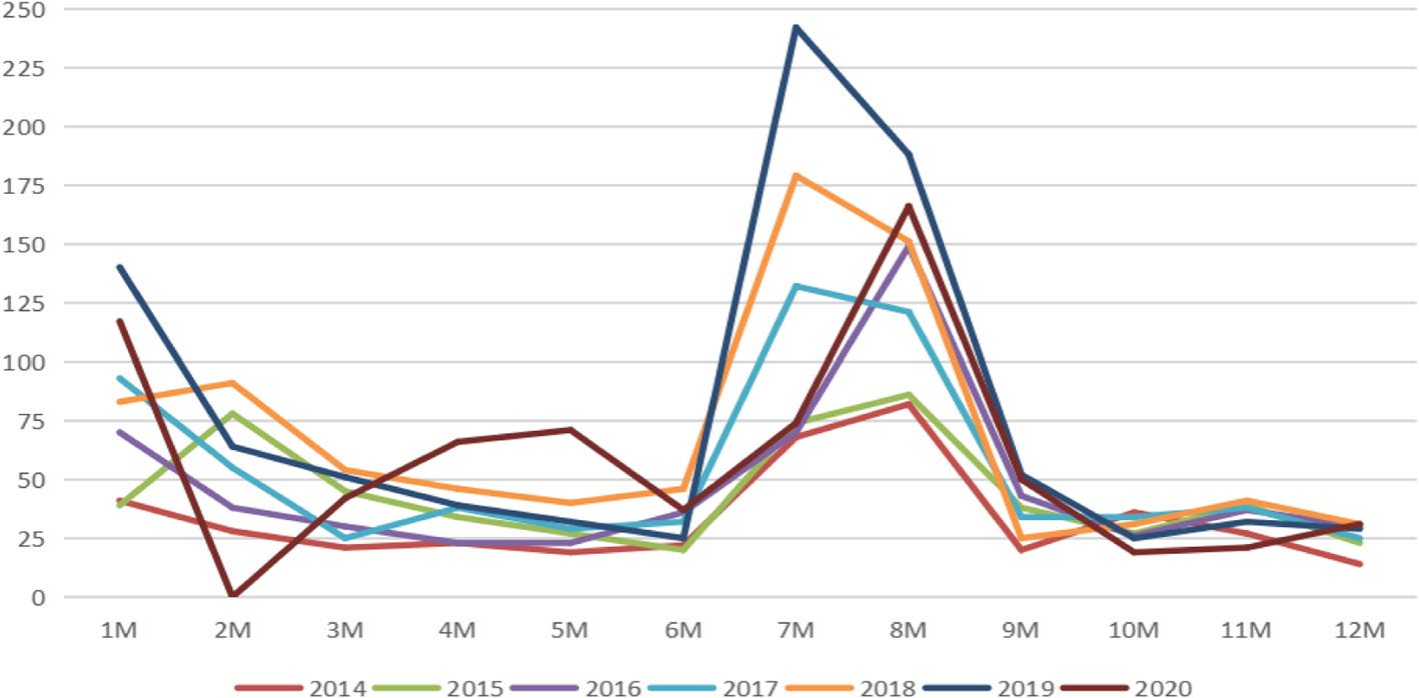
Distribution diagram of the number of strabismus in surgical patients from 2014 to 2020

We also found that most patients were operated on in our hospital in January–February or July–August (Fig. 2), which was related to the fact that most of the patients were students and that holidays also impacted the timing of surgeries.

A limitation of this study is that it was a retrospective case analysis and not a population-based study. Because all samples were collected in the same hospital, the overall population of strabismus cases was not represented. This study could not determine the incidence of strabismus surgery in this region. Because the geographical range was narrow and most of the patients were from the Chaoshan area, the findings of the study cannot be generalized. Further population-based studies are needed to confirm the overall incidence of strabismus and the proportion of strabismus surgery in China. Despite these limitations, the results do reflect the real situation of strabismus in our eye center, and the value of this study lies in the large number of patients involved and the long time span. In the future, we still need to recruit large populations to investigate whether these changes in distribution are universal and whether the prevalence of strabismus in China has changed.

## Conclusions

By analyzing the age at surgery, type of strabismus, and duration of surgery of strabismus patients who underwent surgical intervention from 2014 to 2020, we found that more patients who underwent surgery had constant exotropia than any other strabismus but that its proportion decreased year by year. The proportion of constant exotropia patients younger than 12 years old who received surgical intervention has been increasing in recent years. The proportion of intermittent exotropia patients who underwent surgery has been increasing in recent years; the proportion of such patients younger than 12 years old has increased every year since 2016. The decrease in constant exotropia patients older than 12 years old who underwent surgery may be related to the increase in intermittent exotropia patients younger than 12 years old who have undergone surgical intervention. Therefore, efficient screening should continue. Early detection, early treatment, and restoring normal visual function to children are our goals.

## Data Availability

The datasets generated and/or analysed during the current study are not publicly available due individual privacy but are available from the corresponding author on reasonable request.
